# Supplementary material for: Exploring the Therapeutic Potential of Estrogen-Related Receptor γ Inverse Agonists in Atopic Dermatitis-like Lesions
Source: Int J Mol Sci. 2025 Jul 20;26(14):6959. doi: 10.3390/ijms26146959 (PMC12295363; doi:10.3390/ijms26146959)
Supplement: Supplementary file 1 [file ijms-26-06959-s001.zip › ijms-3746751-supplementary.pdf]

Supporting information

Exploring the Therapeutic Potential of Estrogen-Related Receptor γ Inverse Agonists in Atopic Dermatitis-like Lesions

Ju Hyeon Bae [1], Sijoon Lee [1], Jae-Eon Lee [1], Sang Kyoon Kim [1], Jae-Han Jeon [2] and Yong Hyun Jeon [1,*]

[1] Preclinical Research Center, Daegu-Gyeongbuk Medical Innovation Foundation (K-MEDIhub), 80 Cheombok-ro Dong-gu, Daegu 41061, Republic of Korea; wngus7@kmedihub.re.kr (J.H.B.); sjlee1013@kmedihub.re.kr (S.L.); koof12@kmedihub.re.kr (J.-E.L.); ksk1420@kmedihub.re.kr (S.K.K.)

[2] Department of Internal Medicine, School of Medicine, Kyungpook National University Chilgok Hospital, Kyungpook National University, 807 Hoguk-ro, Buk-gu, Daegu 41404, Republic of Korea; ggoloo@hanmail.net

* Correspondence: jeon9014@gmail.com or jeon9014@kmedihub.re.kr; Tel.: +82-10-2455-6046

Materials and Methods

Animals

Five-week-old male BALB/c mouse were purchased from Orient Bio Inc. (Seongnam, South Korea) and housed under semi specific pathogen-free conditions at 24±2°C with a 12h high-dark cycle. All animals were housed at the Institute of Laboratory Animal Center, Daegu-Gyeongbuk Medical Innovation Foundation. All procedures including animals were accepted in conformance with the guidelines of the Laboratory Animal Center, Daegu-Gyeongbuk Medical Innovation Foundation (Approval Number: KMEDI-23047204-00).

Cells

Human keratinocytes (HaCaT cells), provided by cell line service, were cultured in Dulbecco's Modified Eagle Medium (DMEM) with 10% FBS and 1% penicillin-streptomycin at 37℃ in a 5% CO_2 incubator.

Cell viability Assay

Cell viability was analyzed sing a Cell Counting Kit-8 (Dojindo molecular technolo-gies, MD, USA). HaCaT cells ($1×10^4$ cells/ml) were seeded in 96-well plates and treated with either different concentration of DN200434 and with or without TNF-α/IFN-γ (10ng/ml) for 24h. At the indicated time points, CCK-8 (10 μL/well) reagents were added to the cells, followed by further incubation at 37°C for 90 min. The absorbance at 450nm was measured using a plate reader (BioTek instruments, Winooski, USA).

qRT-PCR and IgE assay

RNA from HaCaT cells and AD dorsal skin tissues was extracted and cDNA was synthesized

using the ReverTraAce™ qPCR RT Kit (Code No. FSQ-101), followed by evaluation of ERRγ, IL-6, and TNF-α mRNA expression with quantitative real-time PCR. PCR conditions were 95°C preincubation, maintained for 10 minutes, then 1 cycle of 95°C denaturation for 15 seconds, 55°C annealing for 15 seconds, and 72°C polymerization for 10 seconds. A total of 45 cycles were repeated. Advanced Relative Quantification analysis was used to confirm the structure of the expected quantitative real-time PCR product, and the variability of other gene amplification was calibrated by relative comparison with GAPDH expression.

Irrespective primer information of ERRγ, IL-6, and TNF-α was described as following.

Primer list

Gene	Forward	Reverse
ERRγ	CAG ACG CCA GTG GGA GCT A	TGG CGA GTC AAG TCC GTT CT
IL-6	CTGGAGTCACAGAAGGAGTGG	GGTTTGCCGAGTAGATCTCAA
TNF-α	TGGGAGTAGACAAGGTACAACCC	CATCTTCTCAAAATTCGAGTGACAA
MDC	GATTACGTCCGTTACCGTCTG	TATCCCTGAAGGTTAGCAACAC
RANTES	TATTTCTACACCAGTGGCAAGT	CCCGAACCCATTTCTTCTCT
TARC	GAGTACTTCAAGGGAGCCATTC	TGCCCTGCACAGTTACAAA
IL-8	ACTGAGAGTGATTGAGAGTGGAC	AACCCTCTGCACCCAGTTTTC
GAPDH(m)	AAC TTT GGC ATT GTG GAA GG	ACA CAT TGG GGG TAG GAA CA
GAPDH(h)	AGG TCG GAG TCA ACG GAT TT	TGA CGG TGC CAT GGA ATT TG

Serum IgE was determined by mouse IgE ELISA kits (Biolegend, San Diego, California, USA). All procedures were performed following the manufacturer's instructions.

Western blot

For the extraction of total protein, HaCaT cells were pretreated with DN200434 for 1h and then stimulated with TNF-α/IFN-γ (10ng/ml) for 30min in a CO_2 incubator at 37°C and washed twice with phosphate-buffered saline (PBS). Cell pellets were lysed using

radioimmunoprecipitation assay (RIPA) buffer (Thermo Fisher Scientific, IL, USA) containing protease and phosphatase inhibitor cocktail kit (Thermo Fisher Scientific). The lysed cells were briefly vortexed at intervals and subsequently centrifuged at 13,000 × g at 4°C. The protein sample was quantified with the bicinchoninic acid (BCA) protein assay kit (Thermo Fisher Scientific). Equal amounts of proteins were loaded onto 10% sodium dodecyl sulfate polyacrylamide gel electrophoresis and transferred onto PVDF membranes (Millipore, Billerica, MA, USA). The membrane was blocked with 5% skim milk in Tris-buffered saline (TBS) containing Tween-20 (TBS-T) for an hour and probed with the respective primary antibodies in 5% BSA overnight at 4°C. Following incubation, the membrane was probed with horseradish peroxidase (HRP)-conjugated secondary antibodies for an hour at room temperature. The membrane was washed thrice with TBS-T and the signal was visualized using an enhanced chemiluminescence (ECL) detection reagent (GE Healthcare Life Sciences, Pittsburgh, PA, USA). The primary antibodies used were as follows: pAKT (Santa Cruz, working dilution 1:500), pERK (Cell signaling; dilution 1:1000), p-p38 (Cell signaling; dilution 1:1000), p-NFκB (Cell signaling; dilution 1:1000)and β-actin (Cell Signaling; dilution 1:5000). HRP-conjugated secondary antibodies used were as follows: anti-mouse (Cell Signaling) and anti-rabbit (Cell Signaling). Control skin lesion and DNCB-induced AD lesion were collected to determine the status of ERRγ expression. Western blotting analysis was accomplished following the previously described methods.

***In vivo* study**

After anesthetizing mice with isoflurane, the hair on the back was removed with a depilator, and the microwounds on the skin were left for 24 hours to heal naturally. Afterwards, the experimental groups were divided into five groups; (1) Control group, (2) 2,4-

dinitrochlorobenzene (DNCB)-induced AD, (3) DNCB-induced AD+10mg/kg prednisone, (4) DNCB-induced AD+1mg/kg DN200434, and (5) DNCB-induced AD+10mg/kg DN200434. DNCB dissolved in a 1% concentration of solvent (Acetone : Olive oil = 3:1) was topically applied to the back of mice once a day for 1 week, and then 0.5% DNCB was administered 3 times a week from 8 to 14 days. Either DN200434 or prednisone was administered orally every day for 7 days starting from the 7th day following the induction of DNCB-induced AD. The skin thickness of mice was measured every 3 days during induction of DNCB-induced AD using a digital caliper. The marked back skin area was measured three times, and the average value was recorded. In case of the measurement of spleen index, each spleen was weighed to calculate the spleen index: spleen index = spleen weight/body weight ×100%.

Skin dermatitis severity

The severity of dermatitis was assessed macroscopically according to the Eczema Area and Severity Index (EASI) scoring system: 0, no symptoms; 1, mild symptoms; 2, moderate symptoms; and 3, severe symptoms. The severity of dermatitis was evaluated by the naked eye of three blind examiners. The sum of the individual scores was defined as the dermatitis score for erythema/hemorrhage, edema, excoriation/erosion and scaling/dryness [18].

Histopathological evaluation of atopic dermatitis-like skin lesions

To evaluate histopathological characteristics, dorsal skin from mice was fixed with 4% (w/v) paraformaldehyde for 24 h at room temperature and embedded in paraffin blocks. Sections (5 μm) were cut using a microtome, then stained with hematoxylin and eosin (H&E). Images were then captured under a microscope (Olympus, BX53, Melville, NY, USA). In the H&E slide, the severity of the hyperplasia of epidermis, inflammation of dermis, hyperkeratosis, folliculitis of hair follicles was scored from 0 to 4: 0 (normal), 1 (mild), 2 (slight), 3 (moderate), and 4

(severe). In toluidine blue stained slides, three fields were randomly selected, and the mast cells were counted. All histopathological evaluation were conducted in a blinded manner by three pathologists with peer review.

Statistical analysis

All statistical analysis were performed using PRISM software version 10. Statical significances were determined using an unpaired Student *t* test. $p < 0.05$ was considered to indicate a statistically significant difference.
